# Book review of “Imaging in Cellular and Tissue Engineering” edited by Y Hanry Yu and Nur Aida Abdul Rahim

**DOI:** 10.1186/1475-925X-13-84

**Published:** 2014-06-23

**Authors:** Eric Michael Brey

**Affiliations:** 1 Pritzker Institute of Biomedical Science and Engineering, Illinois Institute of Technology, 3255 S Dearborn St, Chicago, IL 60616, USA; 2 Research Service, Hines Veterans Administration Hospital, Hines, IL, USA

**Keywords:** Tissue engineering, Regenerative medicine, Imaging, Biomaterials, Assessment

## Abstract

This article is a review of the book “Imaging in Cellular and Tissue Engineering” (ISBN-13: 978-1439848036, $149.95, 298 Pages, 114 Illustrations) edited by Y Hanry Yu and Nur Aida Abdul Rahim published by the CRC Press (Taylor&Francis) in 2013. The contents of the book and its relevance to tissue engineering and regenerative medicine are discussed in this invited review.

## Book details

The fields of tissue engineering and regenerative medicine have significant potential to impact the practice of medicine by providing new options for the reconstruction and replacement of damaged tissues and organs. While this potential is well-known the clinical impact of these therapies has not been fully realized. A significant issue in the field is the limited ability to appropriately evaluate the structure and function of the tissues. While a number of potential tools exist, biomaterial scaffolds and engineered tissues present unique imaging and assessment challenges for researchers [1]. This issue is recognized by funding agencies [2] and scientists [1], but researchers continue to use methods that provide incomplete or misleading information about their research.

Professor Hanry Yu and Dr. Nur Aida Abdul Rahim provide a timely and important multi-edited text that seeks to address this issue with an “all-encompassing tome” [3] presenting imaging modalities used in cellular and tissue engineering applications. The book, entitled “Imaging in Cellular and Tissue Engineering” and published by the CRC Press (Taylor&Francis) [4], is divided into sections on *in vitro* Applications, *in vivo* Applications and a single chapter section on Data Analysis. The book takes a broad view of cellular and tissue engineering Applications are not limited to engineering of replacement tissues with presentation of imaging techniques that assist with high throughput drug screening, cell and tissue characterization in culture and stem cell tracking *in vivo*. It is this broad view that increases its audience. This book would be beneficial to researchers in many fields including tissue engineering, biomaterials, cell biology and drug development/screening.

While imaging is critical to the evaluation of new therapies, researchers in the fields of tissue engineering and regenerative medicine are typically not trained in imaging sciences. This book provides an overview of many imaging modalities. Fundamentals of the techniques are often described and examples of their application in cell and tissue engineering application provided. While a reader would need to refer to other sources for a more advanced presentation of the underlying physics or implementation of a given technique, the book serves as an excellent starting point for investigators exploring imaging options for their work.

This is a multi-edited book with contributions from a variety of authors. Generally the chapters adhere to a consistent format with an introduction to the imaging technique followed by more detailed descriptions of application in a specific area of biomedical research. While most emphasize applications in cell and tissue engineering, some of the applications describe medical applications outside of these general areas. Any of the chapters could be read independently from the full book. However, most should be used as a first introduction to a particular imaging approach. The book would be particularly beneficial to tissue engineering researchers who may not have strong imaging backgrounds and want to get introduced to specific imaging applications. However, some of the chapters (e.g. *Atomic Force Microscopy for Cell and Tissue Niches* and *Magnetic Resonance Imaging to Monitor Implanted Constructs*) provide significant background and detail to be used as a supplement graduate or advanced undergraduate courses.

Professor Yu, Dr. Abdul Rahim and CRC Press provide a text that has consistent structure and typesetting. The price ($149.95) is consistent with specialized scientific texts.

Overall, the book covers a broad range of imaging modalities with both common and new methods discussed in a clear fashion. While the book would have benefited from chapters on exciting new technologies, such as photoacoustic microscopy, fluorescence tomography and x-ray phase contrast, it accomplishes its goal of serving as a single source summarizing most of the applications of interest to the community. This book would be a useful resource for researchers in the fields of cell and tissue engineering who are looking for background on methods for imaging and assessment.

## Competing interests

The author declares no competing interest with regards to this invited review.

## Author’s contributions

EMB reviewed the book, drafted the manuscript and approved the final version.
